# Supplementary material for: Genome concentration limits cell growth and modulates proteome composition in Escherichia coli
Source: eLife. 2024 Dec 23;13:RP97465. doi: 10.7554/eLife.97465 (PMC11666246; doi:10.7554/eLife.97465)
Supplement: Supplementary file 2. [file elife-97465-supp2.docx]

**Supplementary File 2**

| **Para-meter** | **Biological meaning** | **Unit** | **Description** |
| --- | --- | --- | --- |
| $r_{1}$ | $\frac{bulk transcription rate}{total protein number}$ | ${10}^{-3}$/min | This parameter was estimated as $r_{1}=\theta_{RNAP}{\beta_{mRNA} /T}_{RNAP}$, where $\theta_{RNAP}$ is the number of RNAPs normalized by the number of proteins; $\beta_{mRNA}$ is the fraction of RNAPs engaged in transcription, and $T_{RNAP}$ is the mean mRNA synthesis time. |
| $r_{2}$ | $\frac{bulk translation rate}{total protein number}$ | ${10}^{-3}$/min | This parameter was estimated by $r_{2}=\theta_{ribo}{/T}_{ribo}$, where $\theta_{ribo}$ is the number of ribosomes normalized by the number of proteins, and $T_{ribo}$ is the mean protein synthesis time. |
| $K_{1}$ | Saturation level of DNA with respect to RNAP binding | 1/ $\mu m^{3}$ | This parameter was estimates from $\alpha_{RNAP}=\frac{[Z]}{K_{1}+[Z]}$ , where $\alpha_{RNAP}$ is measured in this work and $\left[ Z \right]_{avg}$, the mean genome copy. |
| $K_{2}$ | Saturation level of mRNA with respect to ribosome binding | 1/ $\mu m^{3}$ | This parameter was estimated as $\alpha_{ribo}=\frac{[X]}{K_{2}+[X]}$ , where $\alpha_{ribo}$ is measured in this work and $[X]$, the mRNA concentration. |
| $\delta$ | mRNA degradation rate | 1/min | $\delta=1/\tau_{L}$, where $\tau_{L}$ is the mRNA lifetime |
| $c$ | $\frac{cell volume}{protein number}$ | ${10}^{-6}\mu m^{3}$ | Cell volumes of different culture conditions were measured in this work. |
| $c'$ | $\frac{cell area}{protein number}$ | ${10}^{-6}\mu m^{2}$ | Cell area of different culture conditions was measured in this work. |
| $[{Z]}_{avg}$ | Average genome concentration  of normal-growing cells | genome/ $\mu m^{3}$ | Estimated by averaging the genome concentration over the cell cycle, see Appendix 1 for details. |
