# Supplementary material for: Genome concentration limits cell growth and modulates proteome composition in Escherichia coli
Source: eLife. 2024 Dec 23;13:RP97465. doi: 10.7554/eLife.97465 (PMC11666246; doi:10.7554/eLife.97465)
Supplement: Supplementary file 3. [file elife-97465-supp3.docx]

**Supplementary File 3**

| **Parameter** | **Unit** | **Initial estimation** | **Optimized parameters** | |
| --- | --- | --- | --- | --- |
|  |  |  | **M9glyCAA**  **(model A)** | **M9glyCAA**  **(model B)** |
| $r_{1}$ | ${10}^{-3}$/min | 1.99 | 1.22 | 1.76 |
| $r_{2}$ | ${10}^{-3}$/min | 20.0 | 22.4 | 22.5 |
| $K_{1}$ | 1/ $\mu m^{3}$ | 1.40 | 1.17 | 1.55 |
| $K_{2}$ | 1/ $\mu m^{3}$ | 622 | 797 | 532 |
| $\delta$ | 1/min | 0.96 | 1.27 | 1.31 |
| $c$ | ${10}^{-6}\mu m^{3}$ | 0.28 | 0.28 | 0.18 |
| $c'$ | ${10}^{-6}\mu m^{2}$ | 0.47 | - | - |
| $X_{ini}$ | ${10}^{2}$/cell | 20.2 | - | - |
| $Y_{ini}$ | ${10}^{6}$/cell | 3.82 | - | - |
| $[{Z]}_{avg}$ | genome  /cell | 1.40 | - | - |
