# Supplementary material for: Genome concentration limits cell growth and modulates proteome composition in Escherichia coli
Source: eLife. 2024 Dec 23;13:RP97465. doi: 10.7554/eLife.97465 (PMC11666246; doi:10.7554/eLife.97465)
Supplement: Supplementary file 6. — The abbreviations kan, cat, and spec refer to gene cassette insertions conferring resistance to kanamycin, chloramphenicol, and spectinomycin, respectively. These insertions are flanked by Flp site-specific recombination sites (frt) that allow the removal of the insertion using Flp recombinase from plasmid pCP20 (Cherepanov and Wackernagel, 1995). [file elife-97465-supp6.docx]

**Supplementary File 6**

| **Identifier** | **Genotype** | **Source** |
| --- | --- | --- |
| CJW2591 | CB15N ∆*bla6 ftsZ*::pBGent-pXyl-ftsZ | BamHI/HindIII fragment was cut from YF1585 and cloned into pBGent. The resulting plasmid was integrated into LS107 (Wang et al., 2001). |
| CJW3673 | CB15N *ftsZ*::pBGentpXyl-ftsZ *parB*::*ecfp-parB* | UV-inactivated ɸCr30 phage lysate was prepared from CJW2591 and transduced into MT190. |
| CJW4823 | CB15N *parB*::*ecfp-parB dnaA*::Ω xylX::pGM2195 | UV-inactivated ɸCr30 phage lysate was prepared from GM2471. First, pGM2195 was transduced into MT190, followed by a second transduction of Ω into the resulting strain to disrupt the endogenous *dnaA* gene. |
| CJW6737 | DH5α/pKFSS1_parS^pMT1^ | parS^pMT1^ was amplified from FH3518 (Nielsen et al., 2006) using oligonucleotides NT157 and NT158, digested with BamHI and KpnI, and cloned into the BamHI/KpnI sites of pKFSS1_2 (Takacs et al., 2018). |
| CJW7019 | MG1655 *rpsB*::*rpsB-msfgfp-kan* | (Gray et al., 2019) |
| CJW7079 | CB15N *rplA(L1)*::*rplA(L1)-mCherry* | CJW7080 was subjected to counterselection on 0.3% xylose. |
| CJW7080 | CB15N *rplA(L1)*::pNPTS138-rplA(L1)-mCherry-rplA(L1)DOWN | The vector pNPTS138 (M. R. Alley, unpublished) was PCR-amplified using oligomers JSG_018 and JSG_019. A ~500-bp fragment of the 3’ end of the *rplA(L1)* gene, and a ~500 bp fragment immediately downstream of the *rplA* gene (rplA(L1)DOWN), were amplified from a CB15N genome template using oligomers JSG_024 and JSG_025, and JSG_020 and JSG_021, respectively. *mCherry* was amplified from MTLS4218 (Thanbichler et al., 2007) and a linker was introduced using JSG_022 and JSG_023. All products were assembled into a plasmid via Gibson assembly. The resulting plasmid, which was verified by sequencing junctions, was transformed into S17-1 and introduced into CB15N by conjugation with selection for kanamycin and nalidixic acid resistance. |
| CJW7339 | MG1655 *hupA*::*hupA-mCherry-kan* | *hupA*::*hupA-mCherry-kan* from CJW5556 (Paintdakhi et al., 2016) was moved into MG1655 by P1 transduction. |
| CJW7374 | MG1655 *hupA*::*hupA-mCherry dnaC*::*dnaC2(ts) mdoB*::*kan* | The kanamycin resistance cassette was excised from CJW7339 by expressing the Flp site-specific recombinase from pCP20 (Cherepanov and Wackernagel, 1995). *dnaC*::*dnaC2(ts) mdoB*::*kan* from FW1957 (Wu et al. 2010) was subsequently moved into this strain by P1 transduction. |
| CJW7411 | MG1655 *glmS1868*::*parS(pMT1)-frt-kanR-frt* | Plasmid backbone was amplified from CJW6919 (Kim et al., 2019) using oligonucleotides AP0039 and AP0040, pMT1 *parS* was amplified from CJW6737 using AP0041 and AP0042, and the fragments were ligated by Gibson assembly. *parS(pMT1)-frt-kanR-frt* was amplified from the resulting plasmid with AP0078 and AP0079. The amplicon was inserted between *glmS* and *pstS* by λ-red mediated recombination (Datsenko and Wanner, 2000). |
| CJW7457 | MG1655 *lacY(A177C) araFGH*::*spec ∆lacI ∆araE araBAD*::*dCas9 galM<PBBa-J23119-sgRNA(oriC)-(S. pyogenes terminator)-(rrnB terminator)>gmpA hupA*::*hupA-mCherry-kan* | *hupA*::*hupA-mCherry-kan* from CJW5556 (Paintdakhi et al., 2016) was moved into SJ_XTL676 by P1 transduction. |
| CJW7473 | CB15N *rplA(L1)*::*rplA(L1)-mCherry ftsZ*::pBGentpXyl-ftsZ | UV-inactivated ɸCr30 phage lysate was prepared from CJW7080 and used for transduction into CJW3673. Tranductants were selected on kanamycin, and then counterselected on 0.3 % xylose. |
| CJW7477 | MG1655 *lacY(A177C) araFGH*::*spec ∆lacI ∆araE araBAD*::*dCas9 galM<PBBa-J23119-sgRNA(oriC)-(S. pyogenes terminator)-(rrnB terminator)>gmpA hupA*::*hupA-mCherry rpoC*::*rpoC-ygfp-kan* | The kanamycin resistance cassette was excised from CJW7457 by expressing the Flp site-specific recombinase from pCP20 (Cherepanov and Wackernagel, 1995). *rpoC*::*rpoC-ygfp-kan* from RLG7470 was subsequently moved into this strain by P1 transduction. |
| CJW7478 | MG1655 *lacY(A177C) araFGH*::*spec ∆lacI ∆araE araBAD*::*dCas9 galM<PBBa-J23119-sgRNA(oriC)-(S. pyogenes terminator)-(rrnB terminator)>gmpA hupA*::*hupA-mCherry rpsB*::*rpsB-msfgfp-kan* | The kanamycin resistance cassette was excised from CJW7457 by expressing the Flp site-specific recombinase from pCP20 (Cherepanov and Wackernagel, 1995). *rpsB*::*rpsB-msfgfp-kan* from CJW7019 was subsequently moved into this strain by P1 transduction. |
| CJW7500 | CB15N *rplA(L1)*::*rplA(L1)-mCherry dnaA*::Ω xylX::pGM2195 | UV-inactivated ɸCr30 phage lysate was prepared from GM2471. A double transduction was performed into CJW7079. |
| CJW7517 | MG1655 *lacY(A177C) araFGH*::*spec ∆lacI ∆araE araBAD*::*dCas9 galM<PBBa-J23119-sgRNA(oriC)-(S. pyogenes terminator)-(rrnB terminator)>gmpA gtrA*::*P58-mCherry-parB(pMT1) parS(pMT1) at ori1 hupB*::*hupB-cfp-kan* | The strain was generated by first moving *gtrA*::*P58-mCherry-parB(pMT1)-cat* (Wiktor et al., 2021) into SJ_XTL676 by P1 transduction. Next, *parS(pMT1)-kan* at *ori1* from CJW7411 was moved into the strain by P1 transduction. The resistance genes were excised by expressing the Flp site-specific recombinase from pCP20 (Cherepanov and Wackernagel, 1995). Finally, *hupB*::*hupB-cfp-kan* from CJW4656 (Gray et al., 2019) was moved into the strain by P1 transduction. |
| CJW7518 | MG1655 *lacY(A177C) araFGH*::*spec ∆lacI ∆araE araBAD*::*dCas9 galM<PBBa-J23119-sgRNA(oriC)-(S. pyogenes terminator)-(rrnB terminator)>gmpA hupA*::*hupA-mcherry ∆relA251*::*kan ∆spoT207*::*cat* | The kanamycin resistance cassette was excised from CJW7457 by expressing the Flp site-specific recombinase from pCP20 (Cherepanov and Wackernagel, 1995). *∆relA251*::*kan* and *∆spoT207*::*cat* were subsequently moved into this strain by two P1 transductions from strain CF1693 (Xiao et al., 1991). |
| CJW7519 | MG1655 *rpoC*::*rpoC-HaloTag-kan* | The strain was generated by first creating *rpoC-HaloTag-kan* amplicon with oligonucleotides JSG_148 and JSG_149 from strain JM41 (Mäkelä and Sherratt, 2020). This amplicon was used to replace the endogenous *rpoC* by λ-red mediated recombination (Datsenko and Wanner, 2000). Finally, *rpoC*::*rpoC-HaloTag-kan* was moved into MG1655 by P1 transduction. |
| CJW7520 | MG1655 *lacY(A177C) araFGH*::*spec ∆lacI ∆araE araBAD*::*dCas9 galM<PBBa-J23119-sgRNA(oriC)-(S. pyogenes terminator)-(rrnB terminator)>gmpA rpoC*::*rpoC-HaloTag-kan* | *rpoC*::*rpoC-HaloTag-kan* from CJW7519 was moved into SJ_XTL676 by P1 transduction. |
| CJW7522 | MG1655 *lacY(A177C) araFGH*::*spec ∆lacI ∆araE araBAD*::*dCas9 galM<PBBa-J23119-sgRNA(oriC)-(S. pyogenes terminator)-(rrnB terminator)>gmpA hupA*::*hupA-mCherry ΔrecA*::*kan* | *recA* was replaced by *kan* using oligonucleotides JSG_173 and JSG_174 in MG1655 by λ-red mediated recombination (Datsenko and Wanner, 2000). The kanamycin resistance cassette was excised from CJW7457 by expressing the Flp site-specific recombinase from pCP20 (Cherepanov and Wackernagel, 1995) and *∆recA*::*kan* was subsequently moved into the strain by P1 transduction. |
| CJW7527 | MG1655 *lacY(A177C) araFGH*::*spec ∆lacI ∆araE araBAD*::*dCas9 galM<PBBa-J23119-sgRNA(ftsZ)-(S. pyogenes terminator)-(rrnB terminator)>gmpA rpoC*::*rpoC-HaloTag-kan* | *rpoC*::*rpoC-HaloTag-kan* from CJW7519 was moved into SJ_XTL229 by P1 transduction. |
| CJW7528 | MG1655 *rpsB*::*rpsB-HaloTag-kan* | The strain was generated by first creating *rpsB-HaloTag-kan* amplicon with oligonucleotides JSG_157 and JSG_158 from strain JM41 (Mäkelä and Sherratt, 2020). This amplicon was used to replace the endogenous *rpsB* by λ-red mediated recombination (Datsenko and Wanner, 2000). Finally, *rpsB*::*rpsB-HaloTag-kan* was moved into MG1655 by P1 transduction. |
| CJW7529 | MG1655 *lacY(A177C) araFGH*::*spec ∆lacI ∆araE araBAD*::*dCas9 galM<PBBa-J23119-sgRNA(oriC)-(S. pyogenes terminator)-(rrnB terminator)>gmpA rpsB*::*rpsB-HaloTag-kan* | *rpsB*::*rpsB-HaloTag-kan* from CJW7528 was moved into SJ_XTL676 by P1 transduction. |
| CJW7530 | MG1655 *lacY(A177C) araFGH*::*spec ∆lacI ∆araE araBAD*::*dCas9 galM<PBBa-J23119-sgRNA(ftsZ)-(S. pyogenes terminator)-(rrnB terminator)>gmpA rpsB*::*rpsB-HaloTag-kan* | *rpsB*::*rpsB-HaloTag-kan* from CJW7528 was moved into SJ_XTL229 by P1 transduction. |
| CJW7531 | CB15N *rpoC*::*mCherry-rpoC* | Two DNA segments of vector pNPTS138 (M. R. Alley, unpublished) were PCR-amplified using oligomers JSG_134 and JSG_137, and JSG_135 and JSG_136. A ~900 bp fragment upstream of the *rpoC* gene and ~900 bp of the 5’ end of *rpoC* were amplified from a CB15N genome template using oligomers JSG_128 and JSG_129, and JSG_132 and JSG_133, respectively. *mCherry* was amplified from MTLS#4218 (Thanbichler et al., 2007) and a linker was introduced using oligomers JSG_130 and JSG_131. All products were assembled into a plasmid via Gibson assembly. Proper plasmid assembly was verified by sequencing junctions. Plasmid was transformed into S17-1 and introduced into CB15N by conjugation with selection for kanamycin and nalidixic acid resistance. Second homologous recombination was achieved by counterselection on 0.3 % xylose. |
| CJW7535 | CB15N *rpoC*::*mCherry-rpoC dnaA*::Ω xylX::pGM2195 | UV-inactivated ɸCr30 phage lysate was prepared from GM2471. First, pGM2195 was transduced into CJW7531, followed by a second transduction of Ω into the resulting strain to disrupt the endogenous *dnaA* gene. |
| CJW7563 | MG1655 *lacY(A177C) araFGH*::*spec ∆lacI ∆araE araBAD*::*dCas9 galM<PBBa-J23119-sgRNA(ftsZ)-(S. pyogenes terminator)-(rrnB terminator)>gmpA rpoC*::*rpoC-ygfp-kan* | *rpoC*::*rpoC-ygfp-kan* from RLG7470 was moved into SJ_XTL229 by P1 transduction. |
| CJW7564 | MG1655 *lacY(A177C) araFGH*::*spec ∆lacI ∆araE araBAD*::*dCas9 galM<PBBa-J23119-sgRNA(ftsZ)-(S. pyogenes terminator)-(rrnB terminator)>gmpA rpsB*::*rpsB-msfgfp-kan* | *rpsB*::*rpsB-msfgfp-kan* from CJW7019 was moved into SJ_XTL229 by P1 transduction. |
| CJW7569 | CB15N *rpoC*::*mCherry-rpoC ftsZ*::pBGentpXyl-ftsZ | UV-inactivated ɸCr30 phage lysate was prepared from CJW2591 and transduced into CJW7531. |
| CJW7576 | MG1655 *lacY(A177C) araFGH*::*spec ∆lacI ∆araE araBAD*::*dCas9 galM<PBBa-J23119-sgRNA(ftsZ)-(S. pyogenes terminator)-(rrnB terminator)>gmpA hupA*::*hupA-mCherry-kan* | *hupA*::*hupA-mCherry-kan* from CJW5556 (Paintdakhi et al., 2016) was moved into SJ_XTL229 by P1 transduction. |
| GM2471 | CB15N *dnaA*::Ω xylX::pGM2195 | (Gorbatyuk and Marczynski, 2001) |
| LS107 | CB15N ∆*bla6* | (West et al., 2002) |
| MG1655 | *F- lambda- ilvG- rfb-50 rph-1* | (Guyer et al., 1981) |
| MT190 | CB15N *parB*::*ecfp-parB* | (Thanbichler and Shapiro, 2006) |
| RLG7470 | *rpoC*::*rpoC-ygfp-kan* | (Bakshi et al., 2012) |
| SJ_XTL229 | *lacY(A177C) araFGH*::*spec ∆lacI ∆araE araBAD*::*dCas9 galM<PBBa-J23119-sgRNA(ftsZ)-(S. pyogenes terminator)-(rrnB terminator)>gmpA* | (Li et al., 2016) |
| SJ_XTL676 | *lacY(A177C) araFGH*::*spec ∆lacI ∆araE araBAD*::*dCas9 galM<PBBa-J23119-sgRNA(oriC)-(S. pyogenes terminator)-(rrnB terminator)>gmpA* | (Si et al., 2017) |
| YB1585 | CB15N *ftsZ*::pBGST18-pXyl-ftsZ | (Wang et al., 2001) |
