# Supplementary material for: Genome concentration limits cell growth and modulates proteome composition in Escherichia coli
Source: eLife. 2024 Dec 23;13:RP97465. doi: 10.7554/eLife.97465 (PMC11666246; doi:10.7554/eLife.97465)
Supplement: Supplementary file 7. [file elife-97465-supp7.docx]

**Supplementary File 7**

| **Name** | **Sequence** |
| --- | --- |
| AP0039 | ACCCAACTTAATCGCTGTAGGCTGGAGCTGCTTCG |
| AP0040 | ATTAATTGCGTTGCGCAGTTCCCTACTCTCGCATGGG |
| AP0041 | GAGAGTAGGGAACTGCGCAACGCAATTAATGTGAGTTAGC |
| AP0042 | CAGCTCCAGCCTACAGCGATTAAGTTGGGTAACGCCA |
| AP0078 | ATCAAACATCCTGCCAACTCCATGTGACAAACCGTCATCTTCGGCTACTTGCAACGCAATTAATGTGAGTTAGC |
| AP0079 | ACAAACATTAATAACGAAGAGATGACAGAAAAATTTTCATTCTGTGACAGCCCTGATTCCGTGAGGATGC |
| JSG_018 | GCCCGAGGGCACGTGACGCGTCTCCGGATG |
| JSG_019 | ACCTCGGCGATCGCGAACGCCGAAGCTAGCGAATTCGTGGATC |
| JSG_020 | GAATTCGCTAGCTTCGGCGTTCGCGATCGCCGAGGTG |
| JSG_021 | GGCATGGACGAGCTGTACAAGTAAGCCACCCAAGTTTTAGTAAGCG |
| JSG_022 | CGCTTTACTGGGTACGCTTACTAAAACTTGGGTGGCTTACTTGTACAGCTCGTCCATGCC |
| JSG_023 | AACGTTACGCGTCACCGGTCGGCCACCATGGTGAGCAAGGGCGAG |
| JSG_024 | GGCCGACCGGTGACGCGTAACGTTCGAATTGGCGCCGATCGAGCTGATG |
| JSG_025 | CGGAGACGCGTCACGTGCCCTCGGGCACCGGCC |
| JSG_128 | TCCGTAATACGACTCACTTAAGGCCTTGACGATGGTCAAGGTCTTCGTGG |
| JSG_129 | GCCATGTTATCCTCCTCGCCCTTGCTCACCATCTTGGTTCCTTCTGCGGG |
| JSG_130 | TTTTCGCGGGAAACCCCGCAGAAGGAACCAAGATGGTGAGCAAGGGCGAG |
| JSG_131 | TTCAGGACTTCCTGGTTCATGCTGCCGCTGCCGCTGCCCTTGTACAGCTCGTCCATGC |
| JSG_132 | AGCTGTACAAGGGCAGCGGCAGCGGCAGCATGAACCAGGAAGTCCTGAAC |
| JSG_133 | ATGTACAGGCATGCGTCGACCCTCTAAGCATCCGCTTTTCGTTG |
| JSG_134 | CGCAACGAAAAGCGGATGCTTAGAGGGTCGACGCATGC |
| JSG_135 | CACGAAGACCTTGACCATCGTCAAGGCCTTAAGTGAGTCG |
| JSG_136 | TTATCCGTACTCCTGATGATGC |
| JSG_137 | GGGATCGCAGTGGTGAGTAAC |
| JSG_148 | GCCTGGCAGAACTGCTGAACGCAGGTCTGGGCGGTTCTGATAACGAGTAATCGGCTGGCTCCGCTGCT |
| JSG_149 | CATAAAAAAACCCGCCGAAGCGGGTTTTTACGTTATTTGCGGATTAACGAGGATCCCATATGAATATCCTCCTT |
| JSG_157 | GTTCTCAGGATCTGGCTTCCCAGGCGGAAGAAAGCTTCGTAGAAGCTGAGTCGGCTGGCTCCGCTGCT |
| JSG_158 | TTGCCGCCTTTCTGCAACTCGAACTATTTTGGGGGAGTTATCAAGCCTTAGGATCCCATATGAATATCCTCCTT |
| JSG_173 | AAAAAAGCAAAAGGGCCGCAGATGCGACCCTTGTGTATCAAACAAGACGAGTGTAGGCTGGAGCTGCTTC |
| JSG_174 | CAACAGAACATATTGACTATCCGGTATTACCCGGCATGACAGGAGTAAAATGTACAAGAAAGCTGGGTACG |
| NT157 | TATGGATCCTGTTTTTCACCACGCCAATTTCATGG |
| NT158 | TATGGTACCGAGGTTGAAAAGCGTGGTG |
