# Supplementary material for: Genome concentration limits cell growth and modulates proteome composition in Escherichia coli
Source: eLife. 2024 Dec 23;13:RP97465. doi: 10.7554/eLife.97465 (PMC11666246; doi:10.7554/eLife.97465)
Supplement: Supplementary file 8. [file elife-97465-supp8.docx]

**Appendix 1 – Supplementary File 1**

| **Symbol** | **Parameter** | **Growth condition** | | | **Source** |
| --- | --- | --- | --- | --- | --- |
|  |  | **M9glyCAAT** | **M9gly** | **M9ala** |  |
| $A$ | Mean cell area (μm^2^) | 2.48 | 1.51 | 1.40 | Measured in this work |
| $w$ | Mean cell width (μm) | 0.80 | 0.69 | 0.62 | Measured in this work |
| $L$ | Mean cell length (μm) | 3.25 | 2.44 | 2.51 | Measured in this work |
| $V$ | Mean cell volume (μm^3^) | 1.50 | 0.83 | 0.70 | Estimated from cell length and width |
| $V_{ini}$ | Cell volume at birth (μm^3^) | 1.08 | 0.60 | 0.50 | $V_{ini}=V/(2log2)$ for exponential-growing population |
| $\lambda$ | Normalized growth rate (1/min) | 0.0173 | 0.0069 | 0.0039 | Measured in this work |
| $\tau_{DB}$ | Doubling time (min) | 40 | 100 | 175 | Estimated from $\lambda$ |
| $\left[ Z \right]_{avg}$ | Average DNA concentration  under balanced growth (genome/μm^3^) | 1.40 | 1.99 | 2.02 | Calculated from cell cycle averaging of the DNA content and cell volume. |
| $\left[ Z \right]_{ini}$ | DNA concentration  of 1N cells at birth  (genome/μm^3^) | 0.92 | 1.67 | 2.00 | Calculated from $V_{ini}$ with the assumption of initial DNA content = 1 genome. |
| $c$ | Cell volume:protein ratio (10^6^ μm^3^) | 0.283 | 0.339 | 0.530 | See Appendix 1 – Supplementary File 1 and Appendix 2 – Supplementary File 1 for $V$ and $Y$ estimates, respectively. |
| $c'$ | Cell area:protein ratio (10^6^ μm^2^) | 0.469 | 0.616 | 1.061 | See Appendix 1 – Supplementary File 1 for $V$ and $A$ estimates. |
