# Supplementary material for: Genome concentration limits cell growth and modulates proteome composition in Escherichia coli
Source: eLife. 2024 Dec 23;13:RP97465. doi: 10.7554/eLife.97465 (PMC11666246; doi:10.7554/eLife.97465)
Supplement: Supplementary file 9. — This table describes how kinetic constants were estimated from the literature. Parameters Xini, Yini, r1,r2 were used in ordinary differential equation (ODE) simulations. We assumed exponential growth and used the relation Mini=M/2log2, where Mini is the biomass of a newborn cell and M is the average cellular biomass in the population (Koch and Schaechter, 1962). The bulk transcription rate r1 was defined as r1=mRNAsynthesisrateofproteins∈thecell , and the bulk translation rate r2 was defined as r2=proteinsynthesisrateofproteins∈thecell . Values were estimated from a previous study (Bremer and Dennis, 2008). For our estimations, we assumed that the average protein length is 310 amino acids and that the average mRNA length is about 1 kb (Ishihama et al., 2008). [file elife-97465-supp9.docx]

**Appendix 2 – Supplementary File 1**

| **Symbol** | **Parameter** | **Value** | **Source** |
| --- | --- | --- | --- |
| $X$ | Number of mRNAs  (per cell) | 2800 | Estimated from (Bremer and Dennis, 2008) |
| $Y$ | Number of proteins  (10^6^/cell) | 5.29 | Estimated from (Bremer and Dennis, 2008) |
| $X_{ini}$ | Number of mRNAs  for a newborn cell | 2020 | Calculated as  $X_{ini}=X/(2log2)$ |
| $Y_{ini}$ | Number of proteins  for a newborn cell (10^6^) | 3.82 | Calculated as  $Y_{ini}=Y/(2log2)$ |
| $n_{RNAP}$ | Number of RNAPs  in the cell (10^3^) | 5.7 | Estimated from (Bremer and Dennis, 2008) |
| $n_{protein}$ | Number of proteins  in the cell (10^6^) | 5.29 | Estimated from (Bremer and Dennis, 2008) |
| $\theta_{RNAP}$ | Fraction of RNAPs relative to the total number of proteins | 0.00108 | $\theta_{RNAP}=n_{RNAP}/n_{protein}$ |
| $\beta_{mRNA}$ | Fraction of RNAPs involved in mRNA synthesis | 0.18 | Estimated from (Bremer et al., 2003). |
| $T_{RNAP}$ | Mean transcription time to generate one mRNA molecule (min) | 0.33 | Estimated from (Bremer and Dennis, 2008) |
| $r_{1}$ | Bulk transcription rate (10^-3^/min) | 1.99 | $r_{1}=\theta_{RNAP}{\beta_{mRNA} /T}_{RNAP}$ |
| $n_{ribo}$ | Number of ribosomes  in a cell (10^3^) | 26 | Estimated from (Bremer and Dennis, 2008) |
| $n_{protein}$ | Number of proteins  in a cell (10^6^) | 5.29 | Estimated from (Bremer and Dennis, 2008) |
| $\theta_{ribo}$ | Ratio between ribosome number and protein number (10^-3^) | 4.91 | $\theta_{ribo}=n_{ribo}/n_{protein}$ |
| $T_{ribo}$ | Mean translation time for one protein molecule (min) | 0.25 | Estimated from (Bremer and Dennis, 2008) |
| $r_{2}$ | Bulk translation rate  (10^-3^/min) | 20.0 | $r_{2}=\theta_{ribo}{/T}_{ribo}$ |
