# Supplementary material for: Genome concentration limits cell growth and modulates proteome composition in Escherichia coli
Source: eLife. 2024 Dec 23;13:RP97465. doi: 10.7554/eLife.97465 (PMC11666246; doi:10.7554/eLife.97465)
Supplement: Supplementary file 10. — The active fraction of RNA polymerases (RNAPs) and ribosomes, αRNAP and αribo, are given by the formulae αRNAP=ZK1+Z and αribo=XK2+X, where [Z] and [X] are the DNA concentration and the mRNA concentration in the cells, respectively. To infer the parameters K1, K2, we used the values of αRNAP, αribo, [Z], and X of wild-type cells determined in our study and back-calculated the values of K1, K2. [file elife-97465-supp10.docx]

**Appendix 2 – Supplementary File 2**

| **Symbol** | **Parameter** | **Value** | **Source** |
| --- | --- | --- | --- |
| $\alpha_{RNAP}$ | Active fraction of RNAPs | 0.5 | Measured in this work |
| $[Z]$ | DNA concentration  (genome/ μm^3^) | 1.40 | Estimated in Appendix 1 – Supplementary File 1 |
| $K_{1}$ | DNA affinity of RNAPs (genome/ μm^3^) | 1.40 | Calculated as  $K_{1}=\frac{1-\alpha_{RNAP}}{\alpha_{RNAP}}[Z]$ |
| $\alpha_{ribo}$ | Active fraction of ribosomes | 0.75 | Measured in this work |
| $[X]$ | mRNA concentration  (1/ μm^3^) | 1867 | $\left[ X \right]=X/V$ |
| $K_{2}$ | mRNA affinity of ribosomes (mRNA/μm^3^) | 622 | Calculated as  $K_{2}=\frac{1-\alpha_{ribo}}{\alpha_{ribo}}[X]$ |
