# Supplementary material for: Genome concentration limits cell growth and modulates proteome composition in Escherichia coli
Source: eLife. 2024 Dec 23;13:RP97465. doi: 10.7554/eLife.97465 (PMC11666246; doi:10.7554/eLife.97465)
Supplement: Supplementary file 11. — The mRNA degradation data were obtained from an experimental study (Balakrishnan et al., 2022). [file elife-97465-supp11.docx]

**Appendix 2 – Supplementary File 3**

| **Symbol** | **Parameter** | **Value** | **Source** |
| --- | --- | --- | --- |
| $\delta$ | mRNA degradation rate (1/min) | 0.964 | (Balakrishnan et al., 2022) |
| $\tau_{d}$ | mRNA lifetime  (min) | 1.04 | Calculated by $\tau_{d}=1/\delta$ |
| $\tau_{H}$ | mRNA half life  (min) | 0.72 | Calculated by $\tau_{H}=(log 2)/\delta$ |
