# Supplementary material for: Genome concentration limits cell growth and modulates proteome composition in Escherichia coli
Source: eLife. 2024 Dec 23;13:RP97465. doi: 10.7554/eLife.97465 (PMC11666246; doi:10.7554/eLife.97465)
Supplement: Supplementary file 12. [file elife-97465-supp12.docx]

**Appendix 3 – Supplementary File 1**

| Model A | Model B |
| --- | --- |
| $\frac{dX}{dt}=r_{1}\alpha_{RNAP}Y$  $r_{1}=\theta_{RNAP}\beta_{mRNA}/T_{RNAP}$  $\alpha_{RNAP}=\frac{[Z]}{K_{1}+[Z]}$ | $\frac{dX}{dt}=r_{1}^{*}\alpha_{RNAP}Y$  $r_{1}^{*}={[\theta}_{RNAP}+bcc'(Y-Y_{o})] \beta_{mRNA}/T_{RNAP}$  $\alpha_{RNAP}\left( \left[ P \right],\left[ Q \right] \right)=1-\left( \sqrt{f_{1}^{2}+f_{2}}-f_{1} \right)$  $f_{1}\equiv\frac{1+A\left[ Q \right]-B[P]}{2B[P]}$, $f_{2}\equiv\frac{1}{B\left[ P \right]}$  $A\equiv\frac{k_{1}}{k_{-1}+k_{2}}\left( 1+\frac{k_{2}}{k_{3}} \right)$, $B\equiv\frac{k_{1}}{k_{-1}+k_{2}}$  $\left[ P \right]=\frac{1}{c}[\theta_{RNAP}+bcc'(Y-Y_{o})]$  $\left[ Q \right]=n_{Q}[Z]$ |
